# Age and Gender Specific Lung Cancer Incidence and Mortality in Hungary: Trends from 2011 Through 2016

**DOI:** 10.3389/pore.2021.598862

**Published:** 2021-04-30

**Authors:** Lilla Tamási, Krisztián Horváth, Zoltán Kiss, Krisztina Bogos, Gyula Ostoros, Veronika Müller, László Urbán, Nóra Bittner, Veronika Sárosi, Aladár Vastag, Zoltán Polányi, Zsófia Nagy-Erdei, Andrea Daniel, Balázs Nagy, György Rokszin, Zsolt Abonyi-Tóth, Judit Moldvay, Zoltán Vokó, Gabriella Gálffy

**Affiliations:** ^1^ Department of Pulmonology, Semmelweis University, Budapest, Hungary; ^2^ Department of Health Policy and Health Economics, Eötvös Loránd University, Budapest, Hungary; ^3^ MSD Pharma Hungary Ltd., Budapest, Hungary; ^4^ National Korányi Institute of Pulmonology, Department of Pulmonology, Budapest, Hungary; ^5^ Mátraháza Healthcare Center and University Teaching Hospital, Mátraháza, Hungary; ^6^ Pulmonology Clinic, University of Debrecen, Debrecen, Hungary; ^7^ Faculty of Medicine, University of Pécs, Pécs, Hungary; ^8^ RxTarget Ltd., Szolnok, Hungary; ^9^ University of Veterinary Medicine, Budapest, Hungary; ^10^ Department of Tumor Biology, National Korányi Institute of Pulmonology – Semmelweis University, Budapest, Hungary; ^11^ 2nd Department of Pathology, MTA-SE NAP, Brain Metastasis Research Group, Hungarian Academy of Sciences, Semmelweis University, Budapest, Hungary; ^12^ Pulmonology Hospital, Törökbálint, Hungary

**Keywords:** epidemiology, lung cancer, incidence, mortality, age and gender

## Abstract

**Objective:** No assessment was conducted describing the age and gender specific epidemiology of lung cancer (LC) prior to 2018 in Hungary, thus the objective of this study was to appraise the detailed epidemiology of lung cancer (ICD-10 C34) in Hungary based on a retrospective analysis of the National Health Insurance Fund database.

**Methods:** This longitudinal study included patients aged ≥20 years with LC diagnosis (ICD-10 C34) between January 1, 2011 and December 31, 2016. Patients with different cancer-related codes 6 months before or 12 months after LC diagnosis or having any cancer treatment other than lung cancer protocols were excluded.

**Results:** Lung cancer incidence and mortality increased with age, peaking in the 70–79 age group (375.0/100,000 person-years) among males, while at 60–69 age group for females (148.1/100,000 person-years). The male-to-female incidence rate ratio reached 2.46–3.01 (*p* < 0.0001) among the 70–79 age group. We found 2–11% decrease in male incidence rate at most age groups, while a significant 1–3% increase was observed in older females (>60) annually during the study period.

**Conclusion:** This nationwide epidemiology study demonstrated that LC incidence and mortality in Hungary decreased in younger male and female population, however we found significant increase of incidence in older female population, similar to international trends. Incidence rates peaked in younger age-groups compared to Western countries, most likely due to higher smoking prevalence in these cohorts, while lower age LC incidence could be attributed to higher competing cardiovascular risk resulting in earlier mortality in smoking population.

## Introduction

Lung cancer was considered globally as a rare disease until the sharp rise starting from the 1930–40’s in the Western world, that culminated in lung cancer becoming the leading cause of preventable cancer -related death among men by the mid-century, with a notable increase in incidence in the female population as well during the past decades [1], showing a strong positive correlation with its main risk factor, the increase of smoking prevalence [2]. According to Cancer Research United Kingdom, a national cancer information portal, 79% of the incident cases in 2015 could have been prevented with effective health promotion interventions, thus avoiding significant financial and societal burden [3].

Numerous publications have found that lung cancer incidence shows a strong correlation with age, the highest incidence rates are found in elderly people [4]. The United Kingdom Cancer Research portal reported that between 2013 and 2015, more than 40% of incident lung cancer cases were diagnosed in the ≥75 years age group [5]. A recent Canadian study from 2015 by Akhtar-Danesh and colleagues, reported diverse trends for lung cancer incidence depending on age and sex: an increasing incidence was observed in elderly female age groups, whereas a decreasing trend in incidence was found among younger women and in most male age cohorts. Age-specific incidence rates started to increase in the age group of 45–49 years, with the highest rates found in the age group of 85–89 years in men and 80–84 years in women [6]. A study by the American Cancer Society from 2016 found that 53% of cases occur in individuals 55–74 years old and 37% occur over 75 years old in the United States. The highest incidence of lung cancer in men is 585.9 per 100,000 in 85–89 years old, while the highest incidence in women is 365.8 per 100,000 person years (PSYs) in 75–79 years old [7]

Previous publications also found differing trends in lung cancer incidence among male and female patients. A study from 2015 examining European cancer trends reported a significant, 12% decrease in the incidence of lung cancer among European men from 2009 to 2015, while an increase of 9% was found among women in the same time period, concluding that lung cancer incidence rates may exceed that of breast cancer in some EU countries by late 2010s’ [8]. Studies from Europe also concluded that in Western countries, men reached the peak of incidence and mortality for lung cancer, showing decreasing trends, with women approaching the plateau. It is mostly established that these trends are influenced by lifestyle, environment and chiefly, smoking habit rather than biological differences associated with gender [9,10].

Hungary is frequently reported having one of the highest incidence and mortality rates of lung cancer in Europe, though in a recent publication, these rates were revised, using a novel methodology based on NHIF database, thus significantly lower rates were found [11]. In recent years, no comprehensive, age and gender specific lung cancer appraisal was conducted in Hungary. Consequently, the primary goal of our study was to determine the age and gender specific incidence and mortality of lung cancer in Hungary between 2011 and 2016, and to assess the trends in both genders by age cohorts.

## Materials and Methods

### Study Design

The National Health Insurance Fund of Hungary (NHIF) provided the input data used for the in-depth analysis in this retrospective, longitudinal study covering lung cancer patients for the 6-years time period between 2011 and 2016. The NHIF is the principal public-healthcare financing agency in Hungary, with its database covering all patients and the entire care continuum for oncology treatments, with the exception of less common private care visits which are outside the scope of this study. The database thus represents nearly 100% of the Hungarian population (9,957,731 people covered – Hungarian Central Statistician Office (CSO) data, 2012), collecting ID and ICD-10 code information from all in- and out-patient visits, as well the entirety of prescription drugs reimbursed in Hungary. The study license number is *I043/88/2019 (at NHIF)*, and the study ethical approval number issued by the Central Ethical Committee of Hungary is *10338–5/2019/EKU.*

Lung cancer patients (ICD-10 C34) who were diagnosed between January 1, 2011 and December 31, 2016 were included in the study, if they were ≥20 years of age at the time of diagnosis. In order to minimize the risk of miscoding lung cancer, patients were included with a minimum of two incidences of the ICD-10 code C34 within more than 30 but less than 365 days. One occurrence of C34 was also accepted if a patient deceased within 60 days after the first C34 code was registered. Patients with different cancer-related codes 6 months before or 12 months after LC diagnosis or having any cancer treatment other than lung cancer protocols were excluded. A period of 3 years between 2008 and 2010 was considered as a reference period to detect newly diagnosed lung cancer patients in 2011. Each patient was tracked until December 31, 2016 or until the time of death. All data were anonymized prior to data extraction stage, ensuring that only non-identifiable data were used for further analyses. The size of relevant Hungarian populations used for incidence and prevalence calculations were obtained from the publicly available annual reports of the Hungarian CSO [12], while mortality data for the lung cancer population was obtained from the source NHIF database.

The annual numbers of newly diagnosed lung cancer patients are presented as crude numbers (n) by age cohorts. Age specific incidence and mortality rates are expressed as rates per 100,000 person-years. All-cause mortality was expressed as crude numbers by age cohorts as well as age-specific rates per 100,000 person-years. In order to allow for comparison with earlier publications, the European Standard Population (ESP) 2013 [13] age groups weights were used for standardization. The sign “<10” was used for raw numbers below 10, as data protection rules of Hungary forbids their publication. All calculations were run on the exact numbers. The following age- cohorts were used for analysis: 20–39; 40–49; 50–59; 60–69; 70–79, 80–89 and ≥90 (expect expressing detailed age specific rates in Supplementary Tables, where we used 20–29 and 30–39 cohorts instead of 20–39).

### Statistical Analysis

Annual trends were estimated with 95% confidence intervals (95% CI). A block-based bootstrap method, with a fixed block size of 2 was used for time series, as data were not independent. Size of population at risk was calculated by subtracting the number of previously diagnosed lung cancer patients on 1^st^ of January from mid-year population, for each study year. Linear regression was applied to calculate the annual change of the mean age of patients. The outcome was age in years, the explanatory variable was the year. The annual odds ratio of the proportion of males among lung cancer patients were calculated by binomial logistic regression. The outcome was the proportion of males, the explanatory variable was the year. Poisson regression was used to estimate crude incidence and mortality numbers, age-specific incidence and mortality rates, and incidence and mortality rate ratios. The outcome was the number of patients, the offset was the log of the number of patients at risk or the mid-year population, the explanatory variables were the year, age group, gender and their paired interactions. Incidence rate ratios (IRR) were calculated using contrasts. Independent linear models were created for the subgroups (sex, age groups) of each line of the Supplementary Tables. The only explanatory variable was the year. For all males, females and the whole patient population, the outcomes were the rates for 100,000 person-years standardized for the ESP 2013. All calculations were performed with R software, version 3.5.2 (2018-12-20) with package boot version 1.3-20.

## Results

### Crude Numbers

In 2011, 4,522 new male lung cancer cases were registered in the NHIF database, while we found 4,176 incident patients in 2016. The number of female patients increased from 2,636 to 2,828 during the same period (Table 1). Proportion of male patients decreased from 63.17% to 56.69% by the end of the study period. The mean age at diagnosis was 64.51 years for men (SD ± 9.85) and 64.93 years for women (SD ± 11.19) in 2011, increasing steadily to 65.80 years (SD ± 9.41) and 65.99 years (SD ± 10.45), respectively.

**TABLE 1:** Crude incidence and mortality of lung cancer by sex and age between 2011 and 2016. SD: standard deviation.

	Number of newly diagnosed lung cancer patients											
	2011		2012		2013		2014		2015		2016	
	Males	Females	Males	Females	Males	Females	Males	Females	Males	Females	Males	Females
Patients with new LC diagnosis (*n*)	4,522	2,636	4,307	2,617	4,126	2,730	4,227	2,722	4,138	2,843	4,176	2,820
Mean age at diagnosis (year)	64.51	64.93	64.85	65.49	64.89	65.56	65.32	65.54	65.58	65.72	65.80	65.99
Mean ± SD	±9.85	±11.19	±9.83	±11.23	±9.95	±11.06	±9.68	±10.89	±9.48	±10.48	±9.41	±10.45
Age group (*n*)												
20–39	30	22	18	31	36	19	24	26	27	19	17	22
40–49	216	180	212	159	203	157	192	150	153	129	170	129
50–59	1,332	719	1,199	672	1,076	746	1,028	709	944	691	898	644
60–69	1,627	886	1,627	872	1,563	874	1,704	955	1,728	1,075	1,786	1,081
70–79	1,029	540	929	567	955	624	976	572	985	644	988	663
80–89	269	259	306	292	270	276	272	284	276	244	291	245
90≤	19	30	16	24	23	34	31	26	25	41	26	36

	Number of lung cancer patients died											
	2011		2012		2013		2014		2015		2016	
	Males	Females	Males	Females	Males	Females	Males	Females	Males	Females	Males	Females
Number of Patients died (*n*)	3,946	2,099	4,059	2,149	3,956	2,198	3,988	2,295	3,861	2,412	4,088	2,377
Mean age at diagnosis (year)	66.09	67.16	66.30	67.38	66.54	67.51	66.66	67.70	66.97	67.92	67.55	68.64
Mean ±SD	±9.83	±11.22	±9.97	±10.93	±9.66	±11.10	±9.79	±10.73	±9.49	±10.54	±9.37	±10.14
Age group (*n*)												
20–39	<10	<10	13	<10	10	<10	14	<10	11	<10	<10	<10
40–49	134	109	152	89	130	99	135	92	123	74	104	56
50–59	1,023	498	997	507	915	512	860	491	751	480	742	390
60–69	1,411	636	1,457	712	1,500	694	1,559	792	1,535	861	1,653	901
70–79	1,019	539	1,046	507	1,037	548	1,038	563	1,082	631	1,174	684
80–89	329	280	377	298	336	296	348	320	325	314	373	297
90≤	23	31	17	31	28	45	34	29	34	45	38	44

Annual number of lung cancer related deaths were between 3,964 (2011) and 4,088 (2016) for males, and 2,099 to 2,377 for females. The mean age at the time of death also increased from 66.09 (SD ± 9.83) to 67.55 years (SD ± 9.37) at males between 2011 and 2016 and from 67.16 (SD ± 11.22) to 68.64 years (SD ± 10.14) among females during the whole study period.

### Age Specific Incidence Rates

**FIGURE 1:**
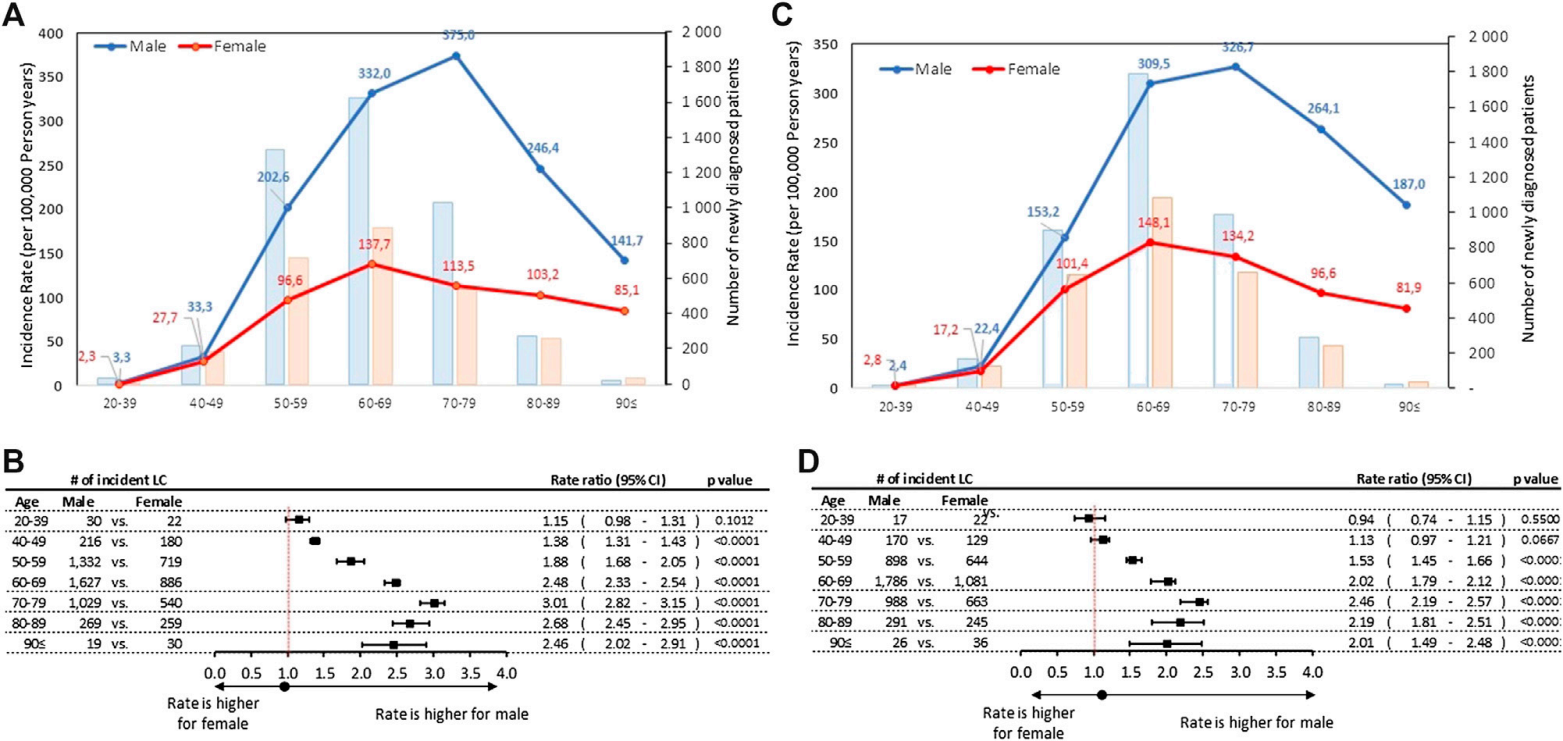
**(A)** Age-specific incidence rates and number of new lung cancer patients by sex in 2011 **(B)** Male-to-female incidence rate ratios by age cohorts in 2011 **(C)** Age-specific incidence rates and number of new lung cancer patients by sex in 2016 **(D)** Male-to-female incidence rate ratios by age cohorts in 2016. CI, confidence interval; LC, lung cancer.

Age-specific incidence rates were higher in men than in women in most age cohorts (Figure 1). Incidence rates for men peaked at 375.0/100,000 PSYs, in the age group of 70–79 years in 2011, declining to 326.7 by 2016, corresponding to a mean annual change of −2.00% (95% CI: 0.16%–2.14%; *p* = 0.0639). Although the crude number of newly diagnosed lung cancer patients was highest in the 60–69 age group in men (1,627 in 2011 and 1,786 in 2016), the highest age-specific incidence rates were observed in the age group 70–79 years (Supplementary Table S1).

Both incidence rates and crude numbers plateaued for women in the age group of 60–69 years (137.7 in 2011 and 148.1 in 2016), without any significant change (*p* = 0.1718) during the 6-years study period. Male-to-female rate ratios were higher among elderly patient: the highest statistically significant male-to-female rate ratio reached 3.01 (95% CI: 2.82–3.15; *p* < 0.0001) in the age group of 70–79 years in 2011, while the lowest was found to be 1.38 (95% CI:1.31-1.43; *p* < 0.0001) in the 40–49 age group in 2011. In the youngest age group (20–39 years) in 2016 the male to female ratio was 0.94, thus we found more incident female patients than male, but the rate ratio was not statistically significant.

**FIGURE 2:**
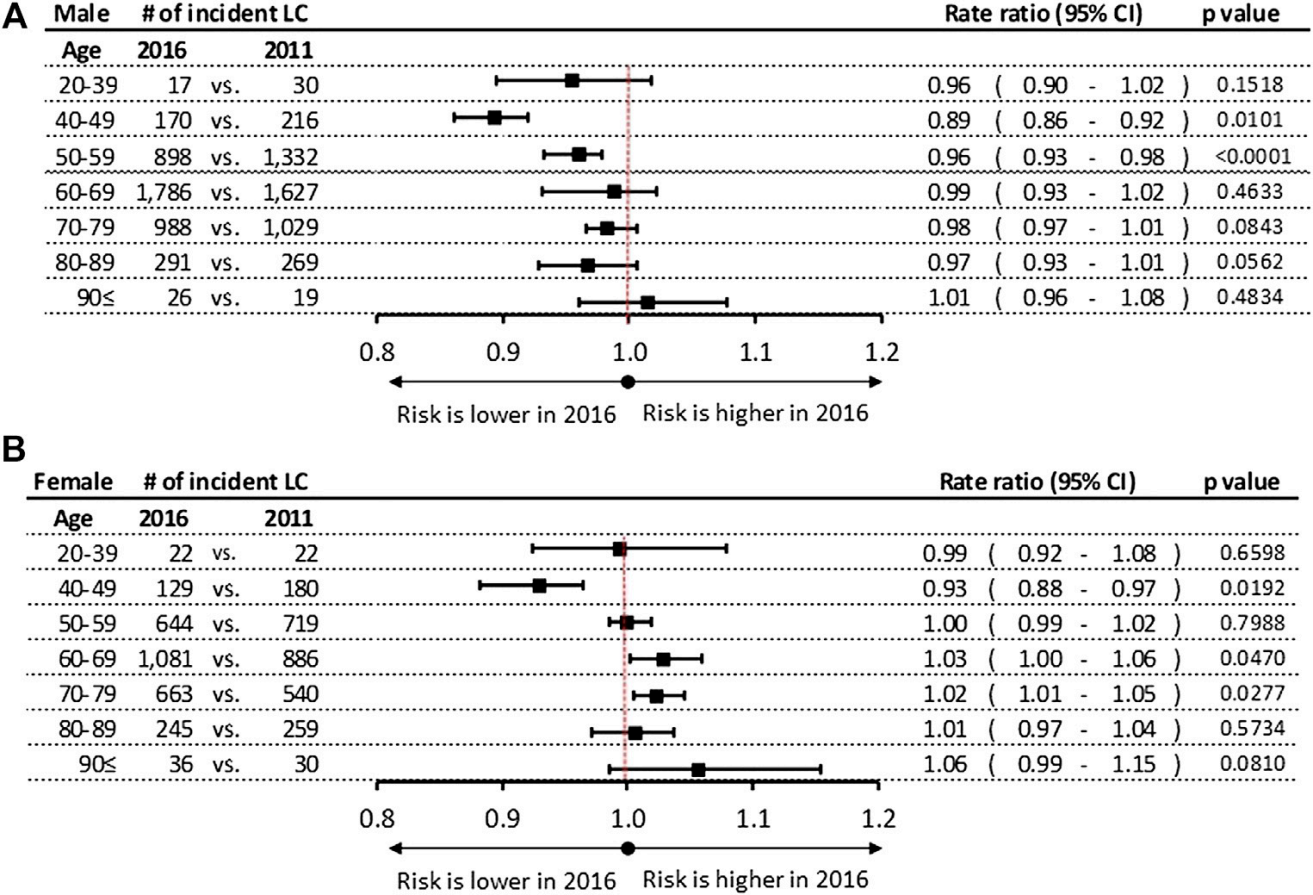
**(A)** Incidence rate ratios by age groups in men in 2016 vs. 2011. **(B)** Incidence rate ratios by age groups in women in 2016 vs. 2011. CI, confidence interval; LC, lung cancer.

Significant differences were found in the incidence rates during 6-years study period among male patients in the age groups of 40–49 and 50–59 years with a 11% (*p* = 0.0101) and 4% (*p* < 0.001) decrease, respectively (Figure 2A). We did not find any significant changes in incidence rates of lung cancer in 2016 vs. 2011 in other age groups in men. In women a significant increase in incidence rates was observed in the age groups of 60–69 years (RR: 1.03; 95% CI: 1.00–1.06; *p* = 0.0470) and 50–59 years (RR: 1.02; 95% CI: 1.01–1.05; *p* = 0.0277), while a 7% decrease was detected in the age group of 40–49 years (RR: 0.93; 95% CI: 0.88–0.97; *p* = 0.0192) (Figure 2B).

### Age Specific Mortality Rates

**FIGURE 3:**
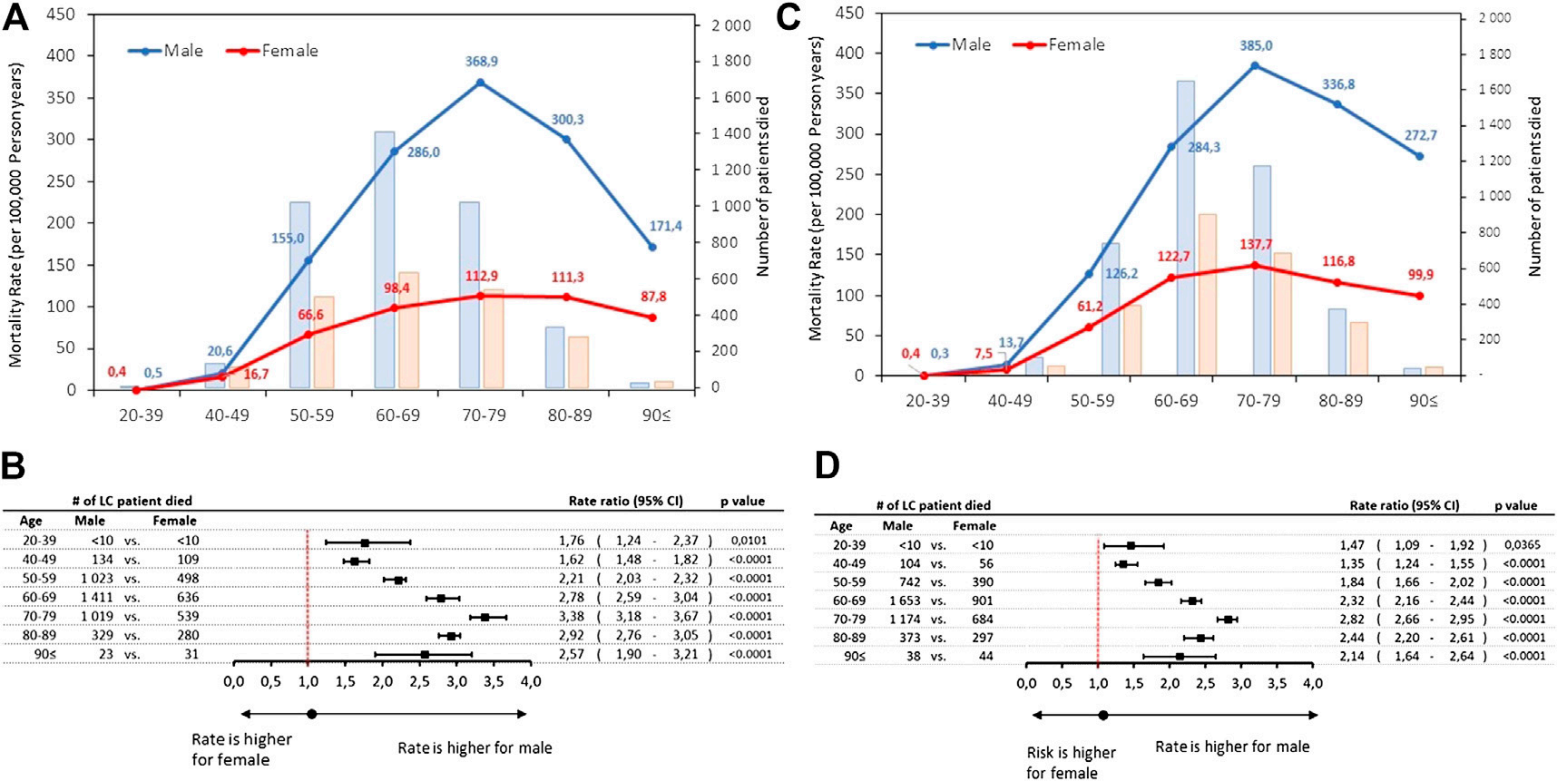
**(A)** Age-specific mortality rates and number of lung cancer death by sex in 2011 **(B)** Male-to-female mortality rate ratios by age cohorts in 2011 **(C)** Age-specific mortality rates and number of lung cancer death by sex in 2016 **(D)** Male-to-female mortality rate ratios by age cohorts in 2016. CI, confidence interval; LC, lung cancer.

The rates of lung cancer mortality were also found to be higher among men, showing a sharp surge from the 50–59 age group and plateauing in the 70–79 years age-group with 368.9 and 385.0/100,000 PSYs in 2011 and 2016, respectively. (Figures 3A,C; Supplementary Table S2). However, the crude number of patients died peaked in 60–69 age group (1,411 and 636 in 2011 increased to 1,653 and 901 in 2016 at males and females, respectively). Male-to-female rate ratios were found to be highest with 3.38 in 2011 (95% CI: 3.18–3.67; *p* < 0.0001) declining to 2.82 (2.66–2.95; *p* < 0.0001) in 2016, respectively (Figures 3B,D).

**FIGURE 4:**
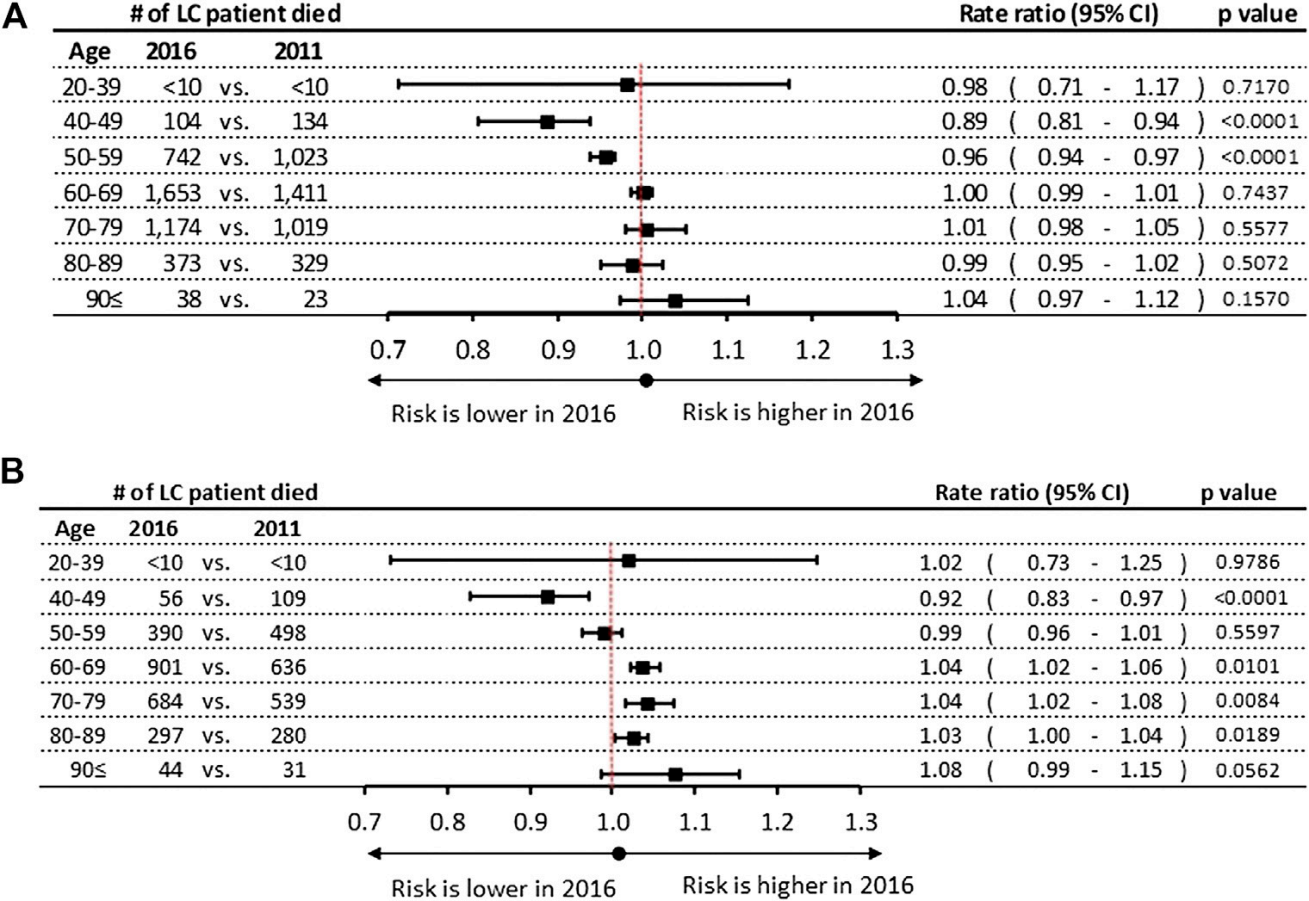
**(A)** Mortality rate ratios by age groups in 2016 vs. 2011 in men **(B)** Mortality rate ratios by age groups in 2016 vs. 2011 in women. CI, confidence interval; LC, lung cancer.

A few statistically significant differences were found in the rates of mortality during the 6 years study period, showing varying trends by gender: we found a decrease among male patients in the age groups of 40–49 and 50–59 years in 2011 (RR: 0.89; 95% CI: 0.81–0.94, *p* < 0.0001 vs. 2016 (RR: 0.96;95% CI: 0.94-0.97, *p* < 0.0001) respectively. (Figure 4A). Inversely increase in mortality rates were found in the female age groups of 60–69, 70–79 and 80–89 years by 4% (95% CI: 1.02–1.06; *p* = 0.0189), 4% (95% CI: 1.02–1.08; *p* = 0.0101), and 3% (95% CI: 1.00–1.04; *p* = 0.0084), respectively (Figure 4B). In contrast, a decrease of 8% was found in female mortality rates in the age group of 40–49 years during the 6-years study period.

## Discussion

This nationwide, retrospective, longitudinal study aimed to provide a comprehensive review of the age and gender specific incidence and mortality of lung cancer in Hungary, using a novel approach in data collection and study design based on the database of the National Health Insurance Fund of Hungary.

The key findings of this database research covering 6 years can be summarized as follows:

1. Age-specific incidence rates of lung cancer were highest in the 70–79 age cohort for men, while it peaked in the 60–69 age group for women.
2. While lung cancer incidence rates showed decreasing trends in most age cohorts for men, for females we found an increase in older age groups.

Differences in lung cancer epidemiology based on gender is a quite thoroughly researched topic, showing similar trends across the developed world, with incidence primarily influenced by prevalence of smoking [14, 15]. Numerous developed countries have reached the peak of their respective tobacco-related lung cancer disease burden, thus incidence and mortality rates started to decline, exhibiting major differences by gender. Historically smoking was always more prevalent among men, leading to higher rates of lung cancer incidence and mortality worldwide [16, 17]. Women took up smoking at a later period, mostly after the Second World War, and their rates of cessation have lagged those of men, leading to a much later peak in lung cancer incidence. In the United States age-standardized incidence rates for males have declined from a peak of 102 in 1984 to 69/100,000 PSYs by 2009, displaying a strong correlation with the decline in smoking prevalence since the 1980’s [18]. However, the trend is showing an opposite curve for females, with age-standardized rates reaching 51/100,000 PSYs, a major increase since 1984, from 39/100,000 PSYs [19, 20].

Similar trends were found in our recently published nationwide NHIF database study where incidence rates among men declined significantly from 115.7 to 101.6 per 100,000 PSYs during the 6-years study period, equivalent to a decrease of 2.35 percent [11]. This trend mirrors the decreasing smoking prevalence rates among Hungarian men, declining from 44% in 1994 to 31% in 2014 [21]. In the current analysis, the highest, 11% reduction of male lung cancer incidence was found in the age group of 40–49 years, although a less pronounced but still significant 4% change was detected in the age group of 50–59 years. In our study, the decrease in the incidence of lung cancer was not significant in older male cohorts (>60 years), which can probably be attributed to the shorter observation period and relatively low patient numbers. Nevertheless, the significant decrease in middle-aged male population may reflect the efficacy of anti-smoking initiatives in Hungary in the last decades [22]. On the other hand, we found increasing rates of lung cancer incidence in the whole female study population, with an annual increase of 2.37%. However, detailed age dependent analysis shows mixed results, the increase was significant in older age groups, while a decrease was detected in the age group of 40–49 years. These findings correspond to a study by Thomas et al. that compared the incidence of non-small cell lung cancer (NSCLC) in lung cancer patients younger and older than 40 years and found decreasing trends between 1975 and 2010 in both cohorts of males, however opposite trends in females lung cancer population, where younger patients had already decreasing trend on incidence, while older (>40) had still increase in incidence rate [23]. Akhtar-Danesh et al. also reported a decrease in the incidence of lung cancer among women in the age group of 50–59 years, and an increase over the age of 60 [6]

Differences in incidence of lung cancer among the genders could be explained by several factors, however, it can be concluded that the most important risk factor for the development of lung cancer is smoking in both genders [14, 24]. Smoking among women has significantly increased since the 1960s, resulting in a consequent increase in the risk of death in the female population [25]. The window of our study most probably covers a period, when we could capture the impact of changing smoking habits due to impact of Hungarian government’s anti-smoking campaign [22]. The European Health Interview Surveys reported smoking prevalence data from Hungary. In 2000, the smoking prevalence of males in the 18–34, 35–64, 65≤ age cohorts were 44.4%, 41.1%, and 13.7% respectively, while by 2 these rates decreased to 36.3% and 36.4% in 18–34 and 35–64 age groups, and slightly increased to 14.1% in the oldest one, showing a 18.2 and 11.2% decrease for the two younger cohorts. On the other hand, we could find a 11.7% decrease at females only in the 18–34 age group (29.0% onto 25.6% during 2000–2009 period), but plateauing trend was seen in the older female population (28.2%–28.8% and 3.4–7.0%) [26]. In their latest report describing smoking habits in Hungary Cselkó et al. reported that smoking prevalence have almost equalized among the genders: the proportion of male smokers showing a steady reduction (32–34%), with the prevalence of smoking stabilizing at 24–25% of the population [27]. This shifting prevalence of smoking is also in line with our findings showing a diminishing difference in the risk of lung cancer incidence and mortality between the genders in all age cohorts. This steady decrease in smoking prevalence at all male age cohorts during the 2000s years could contribute to the decrease of lung cancer incidence in the 2010s, while the peaking smoking prevalence in females could be explained by the increasing lung cancer incidence at most age groups of our study population. The link between smoking and LC is reinforced by a study involving diagnosed lung cancer patients from 2012, that looked at previous smoking status and found that the majority of patients had a history of smoking (79%), including 68% current and 32% former smokers. [28]. The increasing incidence of adenocarcinoma among non-smoking women may also play a role in the narrowing gap between males and females [29]. Other than smoking, several environmental risk factors (air pollution, exposure to asbestos, or radon) may also play a significant role in the changing lung cancer prevalence in both sexes. The impact of an environmental factor beyond smoking may play relevant role in changing prevalence of LC, especially if the size of impact is comparable with smoking habits, however a study examining air pollution trends in Hungary over the last 30 years found that most pollutants have decreased or at least stagnated, hence we believe smoking can be an explanatory factor [30]. Other than controlling exposure to pollutants, the introduction of a comprehensive and rigorously enforced anti-smoking laws of 2011 and 2013, the reduction in retail tobacco selling points, the new multi-media awareness campaign focusing on young generations and the foundation of a Methodological Support Centre for Smoking Cessation at the National Korányi Institute of Pulmonology may have further positive effects in the future by improving lung cancer trends in upcoming decades, in both sexes.

It is observed that the incidence of lung cancer shows strong correlation with age: rates usually start to increase from the age group of 45–49 years, with the highest rates observed in the 85–89 age group among men and in the 80–84 age group among women [31, 32]. In the United Kingdom Cancer Research database, we found similar result: male incidence peaked in 80 or above age cohort close to 600 per 100,000 PSYs, while the rate did not exceed the 100 per 100,000 PSYs till the age of 50 [5]. The peak of incidence was also found in the age group of 80–85 years in the United States as reported by Lung Cancer Statistics, while the highest rate among women was found in the 75–80 age group [7]. The Hungarian male age specific lung cancer incidence rate sharply increases from 40 to 49, exceeding 100 per 100.000 PSYs already by the 50 to 59 age cohorts, peaking in the 60 to 79 age groups, then sharply decreases at 80≤ age groups in both genders. This result is partly different from United Kingdom and United States trends where the peak of incidence was found in older age groups. The United Kingdom Cancer Research database [5] gives detailed tobacco smoking prevalence information, indicating lower smoking prevalence in the younger male and female age groups compared to Hungary but no relevant differences could be found in the older age groups. We could find similar differences in the age specific mortality rates. The earlier peak in the Hungarian lung cancer incidence could be explained by the higher smoking rates, however the lower incidence and mortality rates above the 80 age cohort could be explained by the higher cardiovascular risk of Hungarian population [33], acting as a competing morbidity, resulting in earlier mortality especially among regular heavy smokers [34].

To conclude, our study is the first nationwide investigation describing the age and sex specific incidence and mortality rates of lung cancer in Hungary, showing declining trends in most age groups of males, and an increasing incidence in the older female population in parallel with international trends. The highest incidence rates were found at 70–79 age cohort in males and at 60–69 age cohorts in females, earlier compared to western countries, probably due to the higher competing risk of cardiovascular diseases in elderly people.

There are certain strengths and limitations of our study. The coverage of the whole population in the NHIF database to identify lung cancer patients, the carefully cleaned data, the 6-years-long follow-up period, all provide a solid foundation for drawing conclusions from our analysis. Nevertheless, NHIF database does not contain any data on the staging or the ECOG status of patients, and no laboratory test results were available. Besides, we were not able to examine the competing cause of death in the lung cancer population, and also have no information on the share of active/passive smokers in the population or the rate of non-smoking dependent lung cancer.

Our study emphasizes the needs of putting more focus on the increasing female lung cancer incidence and reinforces the need of more detailed researches to understand more the underlying causes of these negative trends as well as to put more effort on the screening of younger smoker population.

## Data Availability

The original contributions presented in the study are included in the article/Supplementary Material, further inquiries can be directed to the corresponding author.
