# Supplementary figures and images for: Age and Gender Specific Lung Cancer Incidence and Mortality in Hungary: Trends from 2011 Through 2016
Source: Pathol Oncol Res. 2021 Apr 30;27:598862. doi: 10.3389/pore.2021.598862 (PMC8262188; doi:10.3389/pore.2021.598862)

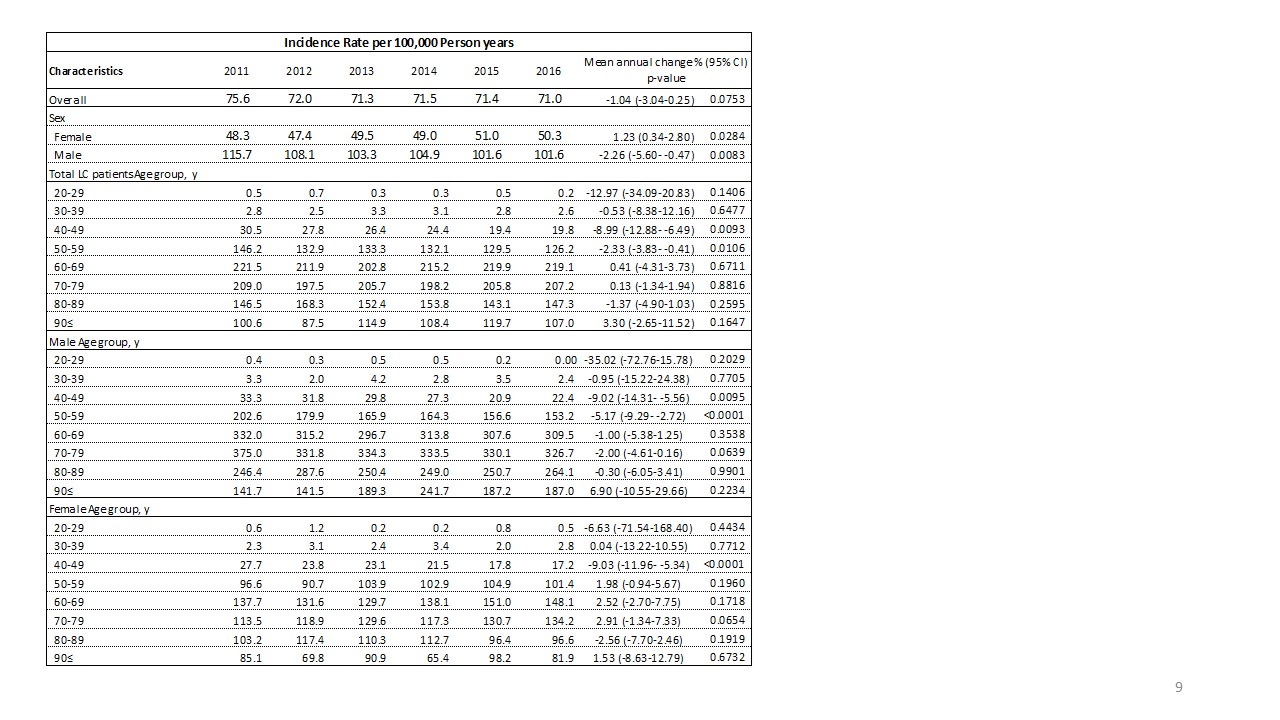

Supplement: Supplementary file 1 [file Image1.JPEG]

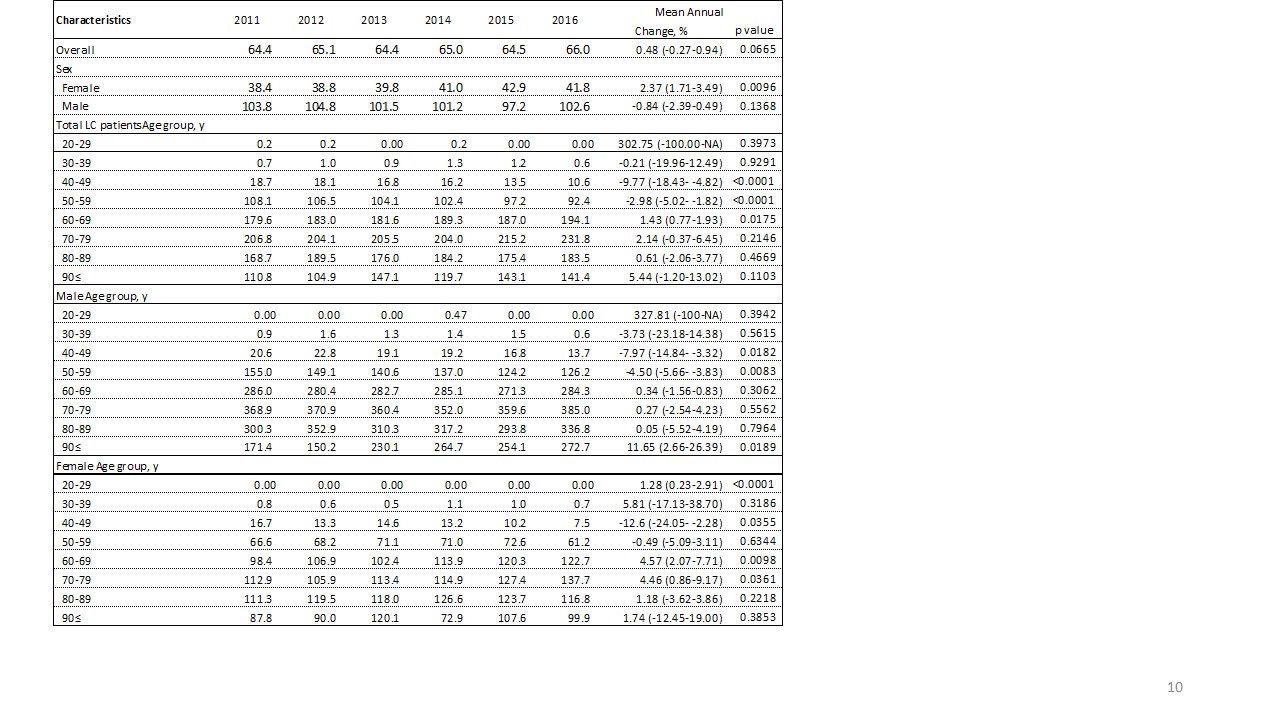

Supplement: Supplementary file 2 [file Image2.JPEG]
